# The role of tides in bottom water export from the western Ross Sea

**DOI:** 10.1038/s41598-021-81793-5

**Published:** 2021-01-26

**Authors:** Melissa M. Bowen, Denise Fernandez, Aitana Forcen-Vazquez, Arnold L. Gordon, Bruce Huber, Pasquale Castagno, Pierpaolo Falco

**Affiliations:** 1 School of Environment, University of Auckland, Auckland, New Zealand; 2 NIWA, Wellington, New Zealand; 3 MetService, Wellington, New Zealand; 4 Lamont-Doherty Earth Observatory, Columbia University, New York, USA; 5 University Parthenope, Naples, Italy; 6 Università Politecnica Delle Marche, Ancona, Italy

**Keywords:** Physical oceanography, Ocean sciences

## Abstract

Approximately 25% of Antarctic Bottom Water has its origin as dense water exiting the western Ross Sea, but little is known about what controls the release of dense water plumes from the Drygalski Trough. We deployed two moorings on the slope to investigate the water properties of the bottom water exiting the region at Cape Adare. Salinity of the bottom water has increased in 2018 from the previous measurements in 2008–2010, consistent with the observed salinity increase in the Ross Sea. We find High Salinity Shelf Water from the Drygalski Trough contributes to two pulses of dense water at Cape Adare. The timing and magnitude of the pulses is largely explained by an inverse relationship with the tidal velocity in the Ross Sea. We suggest that the diurnal and low frequency tides in the western Ross Sea may control the magnitude and timing of the dense water outflow.

## Introduction

About 40% of the ocean volume is Antarctic Bottom Water (AABW) with temperatures and salinities set by the contact with the Antarctic atmosphere^1^. Formation of AABW occurs when dense shelf water descends into the deep ocean in several locations around Antarctica, most notably in the Ross and Weddell Seas and along the East Antarctic Coast. The Ross Sea produces about 40% of the total AABW volume^1^, with the Western Ross Sea contributing about 25%^2^. Changes in AABW properties and formation rate propagate into the global ocean and affect stratification, sea level and heat content^3–5^. Salinity of the high salinity shelf water (HSSW) in the Ross Sea, the precursor of AABW, had been gradually decreasing over the last few decades^6^; however, since 2014 salinity in the western Ross Sea has increased^7^.

**Figure 1:**
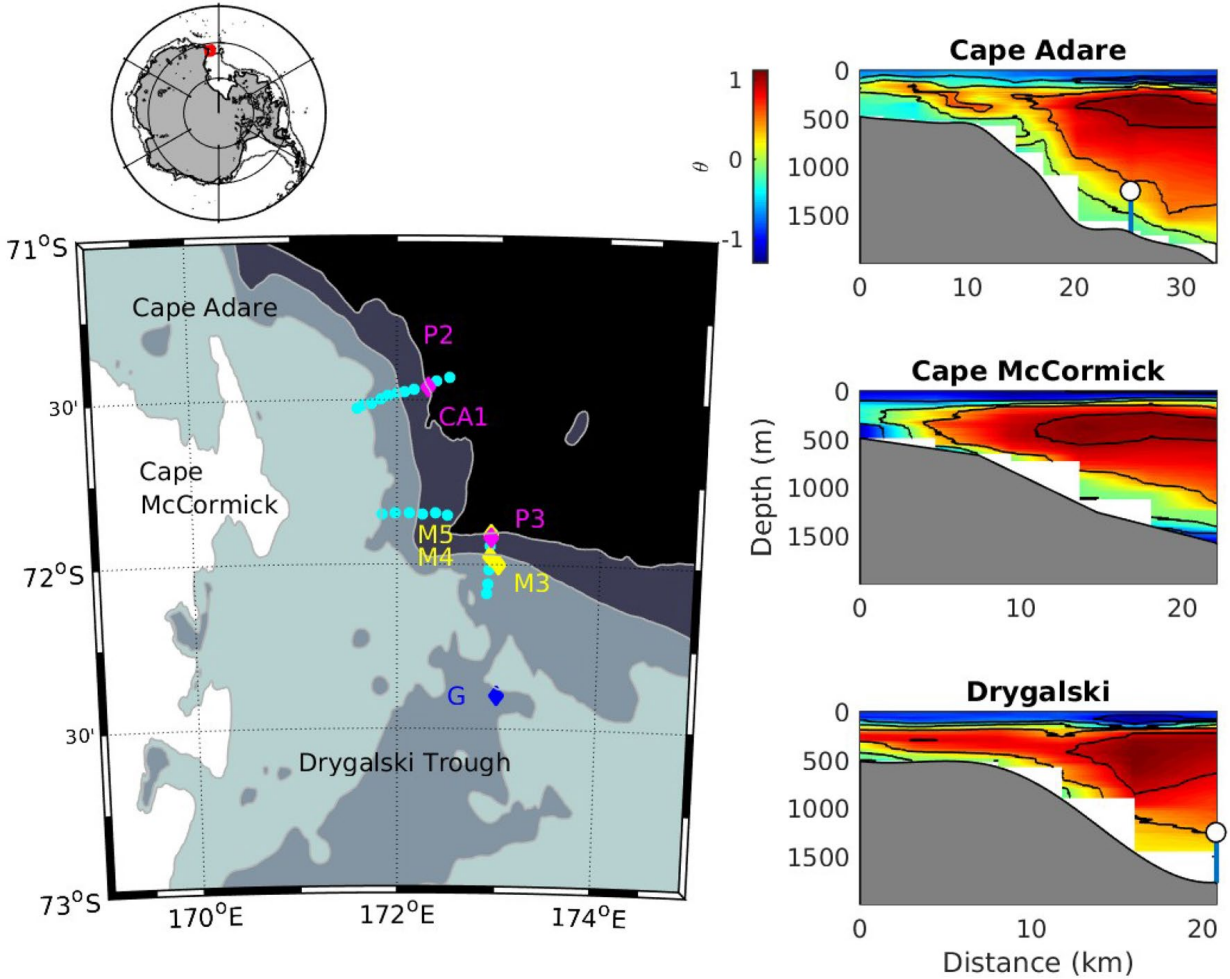
The study site is located on the western side of the Ross Sea (area of regional map shown in red on the upper map). Moorings P2 and P3 were located on the 1750 m isobath off Cape Adare and near the Drygalski Trough respectively (left panel; pink diamonds). The P2 mooring was deployed at the same location as the CA-1 mooring in the CALM experiment. The M3, M4 and M5 moorings were located near the Drygalski Trough during the AnSlope experiment (yellow diamonds). Mooring G in the Drygalski Trough is part of the MORSea Program (blue diamond). Contours are at 500 m, 1000 m, and 1750 m depth. Hydrographic sections were taken in 2018 along three lines perpendicular to the slope (light blue dots). Potential temperatures from the three sections are shown in the panels on the right, with the positions of the P2 and P3 moorings indicated. (Maps produced in Matlab R2015a https://au.mathworks.com/products/matlab.html.).

The water properties of the bottom water from the western Ross Sea are primarily set by dense shelf water plumes exiting the Drygalski and Glomar Challenger Troughs. The dense plumes descend over the continental slope and mix with the warmer Circumpolar Deep Water (CDW)^8–10^ to exit the region as AABW to the northwest at Cape Adare^11^. The steepness of the slope, the Coriolis force and the density of the water all contribute to the trajectory of the dense plumes^10^, with the densest plumes travelling at speeds of over 1 m/s and at angles of 60° to the isobaths^9^. Previous studies have suggested winds may set up pressure gradients that control the release of dense water at the mouths of the troughs. Wind-driven movement of the Antarctic Slope Front (ASF) may modulate the release of dense water from the Drygalski Trough^11^. In the Weddell Sea, the movement of the isopycnals in the boundary current, in response to the wind stress curl in the gyre, may modulate the export of dense water^12^. Winds have also been linked with cross-shelf exchange in the Ross Sea^13^. However, only a few simulations include energetic smaller-scale processes such as mesoscale eddies^14^ and tides^15–18^, and more work is needed to investigate how these contribute to cross-shelf exchange around the Antarctic (Fig. 1).

In the Ross Sea, strong tides may be another control on the release and fate of dense water. Tides in the Ross Sea are primarily diurnal, and therefore modulated by the declination of the sun and the moon, nearly disappearing when the declination of both is zero^19,20^. Observations show tidal advection can shift the ASF to the shelf break of the Drygalski Trough, enabling mixtures of HSSW and CDW to descend over the slope^21^. In the Drygalski Trough itself, CDW is mixed downward to near the bottom when tidal energy is maximum at solstices when solar declination is maximum^22^. Previous studies have also noted a spring-neap modulation of benthic stress, and suggested tidal mixing may influence the dense water properties exiting the trough^15,20^ as well as the dynamics of the dense plumes^23,24^. A series of simulations with different tides in the Drygalski Trough suggest the residual currents causing the outflow of HSSW are maximum at an intermediate tidal strength^17^. Thus, previous studies suggest different responses to the tides: some suggest an increase in HSSW outflow during spring tides^21^, due to tidal advection, others an increase at intermediate tidal strengths due to the residual current^17^.

Here we examine whether the exchange flow may be related to tidal modulation of bottom drag in the Drygalski Trough. Exchange flow varies markedly with the strength of the tides in many estuaries^25,26^ and reduction of tidal velocities allows deep water renewal in some fjords^27^. We investigate how variations of tidal mixing may control the export of bottom water from the Drygalski Trough using observations from moorings adjacent to Cape Adare on the slope and in the Drygalski Trough and from hydrography. We first examine changes in the bottom water between our measurements in 2018 and 2008–2010, when the Cape Adare Long-term Mooring (CALM) experiment took place. We estimate the sources of bottom water at Cape Adare and show that two annual pulses of cold water in 2018 have a contribution of HSSW from the Drygalski Trough. We then show that much of the timing and magnitude of the dense water release from the trough can be related to the modulation of the tidal velocities in the Ross Sea.

## Results

### Interannual and seasonal changes in bottom water at Cape Adare

**Figure 2:**
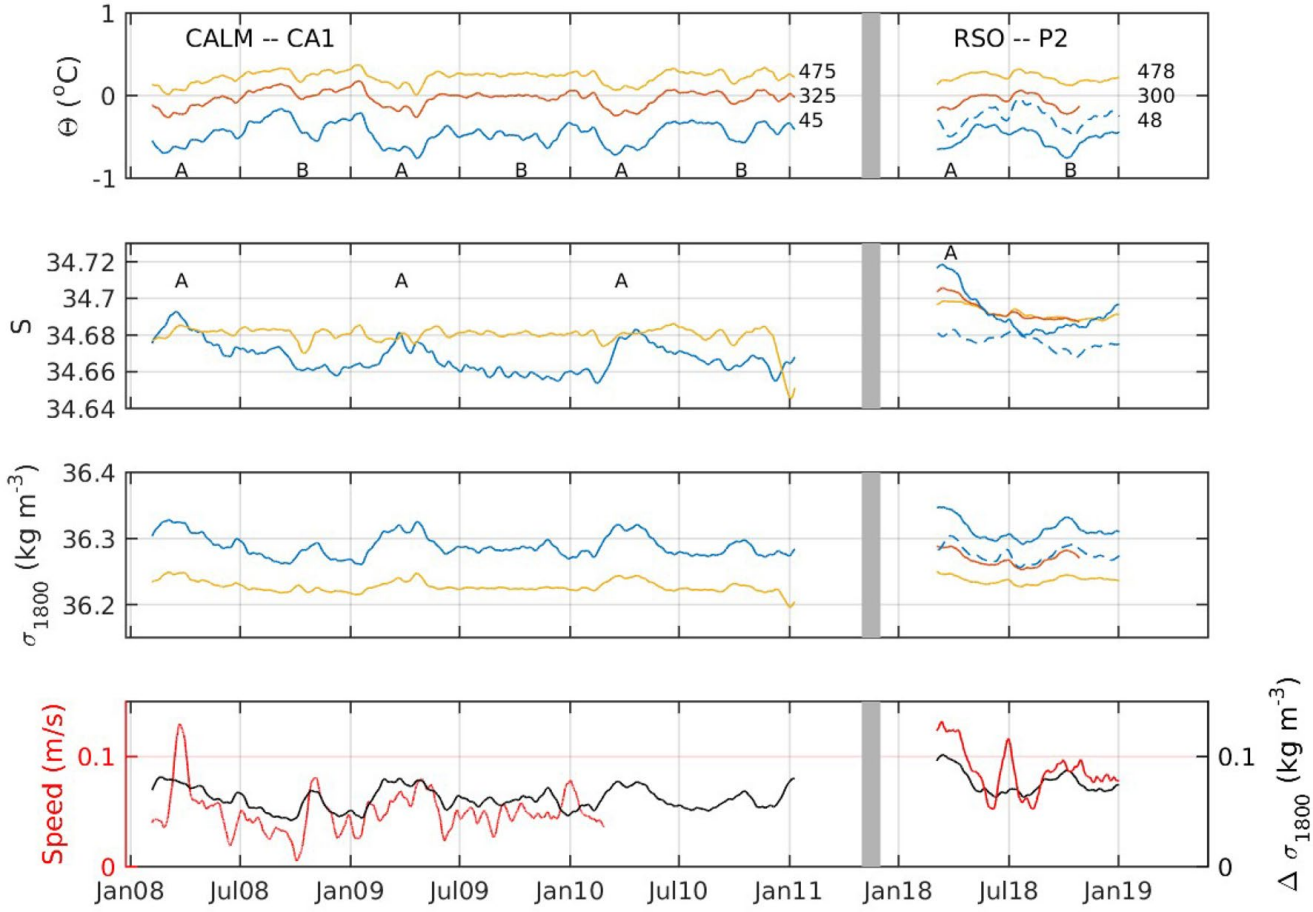
The potential temperature (top panel), salinity (second panel) and potential density at 1800 m (third panel) at the location of the CALM CA-1 mooring and the RSO-P2 mooring. The grey line indicates a discontinuity in time. Sensor height above bottom is shown next to the time series in the upper panel. The letter 'A' marks the cold, salty period every year between March and May. The letter 'B' marks the second cold period during October. The bottom panel shows the density difference between ~ 45 and ~ 475 m above bottom (black line) and the speed of the current at ~ 475 m above bottom (red line). The dashed blue lines show the water properties at the bottom sensor (48 m above bottom) at the RSO P3 mooring on a similar isobath near the Drygalski Trough.

The time series from the CALM CA-1 and RSO-P2 moorings off Cape Adare show an increase in salinities over the bottom 500 m between 2008 and 2018 with the greatest increase near the bottom (Fig. 2). Salinity near bottom reaches a maximum in 2018 that is 0.04 greater than the maximum salinities measured from 2008 through 2010. Temperatures are similar between the two time series. As a result, the average density of the water in the lower 500 m increased in 2018 compared to the earlier time series and there is a slight increase in the density difference between ~ 45 and ~ 470 m above the bottom.

The seasonal cycle near the sea floor at Cape Adare during 2018 is similar to the seasonal cycles observed from 2008 through 2010. A maximum in salinity occurs around March and April coincident with low temperatures (marked with A in Fig. 2). A second period of low temperatures occurs around October (indicated by B) which is not accompanied by a change in salinities. The temperatures around October are particularly low in 2018 compared to 2008–2010.

**Figure 3:**
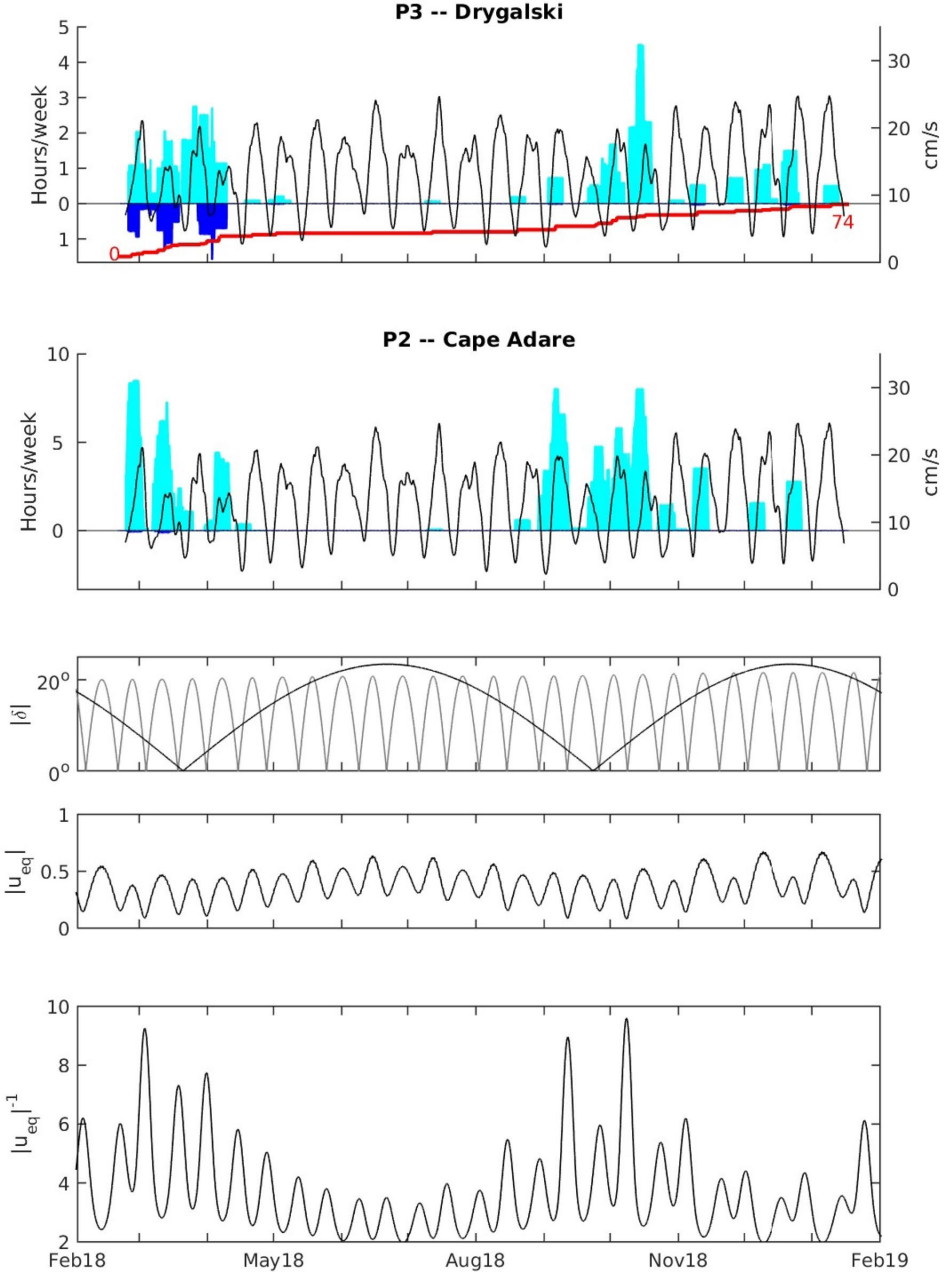
The top two panels show the hours per week that the lowest sensors on each mooring measured water colder than − 1 °C (light blue bars) and also saltier than 34.75 (blue bars pointing downward). The red line shows the cumulative total of plume events at P3 from 0 at the start of the record to 74 at the end. The speed of the tidal flow at 478 m above bottom at P3 averaged over 3 days (black lines) is superimposed over the time of cold water at both moorings. The lower three panels show the absolute value of the declination $(|{\varvec{\delta}}|$) of the sun (black) and moon (gray), the magnitude of the equilibrium tidal velocity averaged over 3 days and the inverse averaged over a week ($\left| {{\varvec{u}}_{{{\varvec{eq}}}} } \right|^{{ - {\mathbf{1}}}}$).

The RSO-P3 mooring, which is situated at a similar isobaths southeast of Cape Adare at the mouth of the Drygalski Trough, measured water that is almost always warmer and less salty than that at Cape Adare (Fig. 2; dashed blue lines). Water travelling along the isobath would take about a week to move between the P2 and P3 moorings, if it travels the average speed measured at the P3 mooring. However, the water at Cape Adare during the two cold pulses in 2018 is too salty to be sourced from the water measured at P3, which is largely coming from further east. We also note that times when cold, salty water is found at Cape Adare are when cold, salty plumes arrive intermittently at P3 (Fig. 3), suggesting HSSW exiting the Drygalski Trough is responsible for both cold periods at Cape Adare.

**Figure 4:**
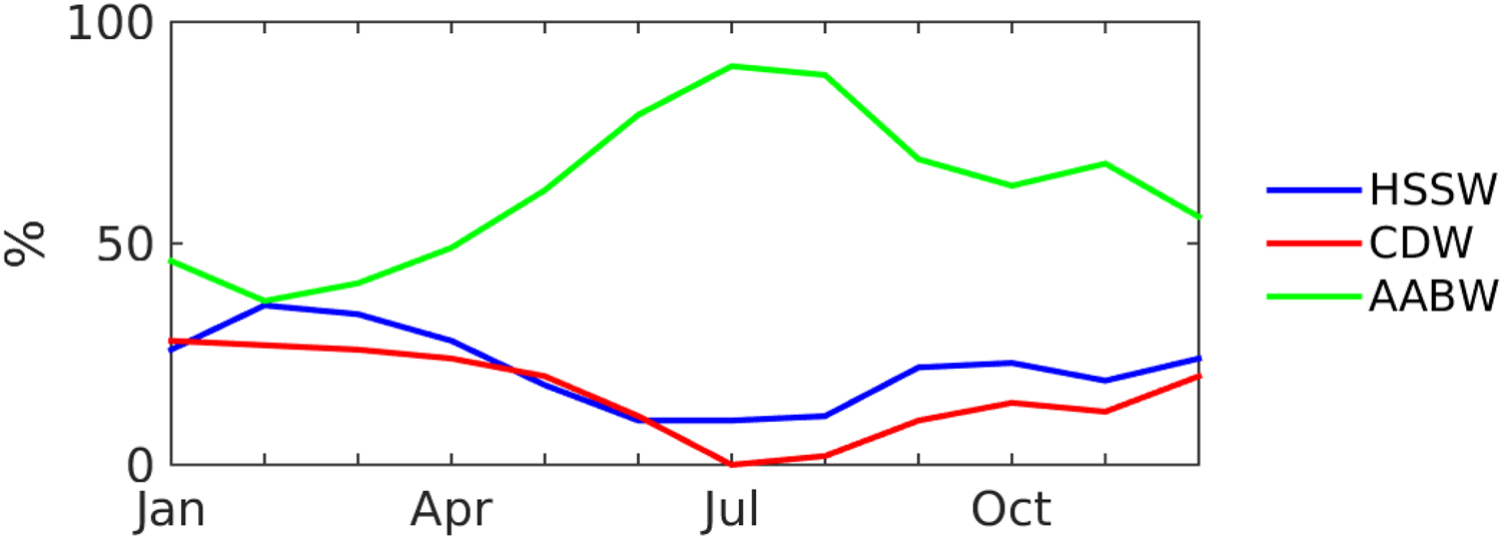
Percentage of water at the bottom sensor of the P2 mooring that can be attributed to HSSW from the Ross Sea (blue), CDW (red) and AABW (green) from further east measured at the bottom sensor of P3.

We investigated what proportion of water from the Drygalski Trough is needed to create the salinity and temperature observed at Cape Adare every month during the RSO deployment (Fig. 4). We use the temperature and salinity of CDW from the hydrography, HSSW properties from the mooring in the Drygalski Trough^22^ and AABW from the water properties at the P3 mooring during each month (see Supplementary Table S1) with all properties taken to the depth of the P2 sensor to find the proportions. The two cold pulses have a higher percentage of HSSW from the Ross Sea than the months before and after (34–36% for February/March and 22–23% for September/October; Supplementary Table S2). The lowest percentages of HSSW occur between May and August (10–18%) when the water properties at Cape Adare are most similar to the AABW coming from the east (estimated as contributing 62–88% of the water at Cape Adare). The seasonal cycle in bottom water salinity at Cape Adare can be explained by the higher salinity in the HSSW observed at Mooring G in the Drygalski Trough in March^22^. The seasonal cycle in salinity suggests an approximately 8-month transit of dense water from formation in the Terra Nova Bay polynya northward to the Drygalski Trough. As noted previously^21^, this time is consistent flow speeds of ~ 0.025 m/s measured at Terra Nova Bay^28^ and the 450 km distance between them and with tracer studies that suggest a transit time of less than a year^29^.

From the hydrographic survey, northward transport of AABW at Cape Adare in February 2018 is 0.5 Sv with 0.3 Sv at neutral densities ${{\varvec{\upgamma}}}_{\mathbf{n}}>28.27$, which corresponds to 20% and 15%, respectively, of the total northward transport of 2.7 Sv. The northward transport from the hydrographic section in 2004 calculated in the same manner is greater: 1.6 Sv of AABW and 1.2 Sv for the higher neutral densities comprising 64% and 48%, respectively, of the total transport of 2.5 Sv. While the total transport estimates are similar, less dense water is observed at the section in 2018 compared to the section in 2004.

### Dense plumes from the Drygalski Trough

HSSW is measured intermittently at the P3 mooring, primarily during March, when water less than − 1 °C and saltier than 34.75 is measured for several hours a week (Fig. 3, top panel). A second period with similarly cold water, but fresher salinities, occurs during the last few months of the year, with the greatest number of events during October. The events at the P3 mooring occur as distinct plumes passing by the mooring with temperatures decreasing and salinities increasing over 20–60 min and returning to their previous values within the next 4–10 h (see Supplementary Fig. S3). These plumes bring dense water very rapidly (within a few hours) from the trough down the slope. We did not expect to see dense plumes at this mooring: the M5 mooring during the AnSlope experiment was at a similar location and had almost no instances where HSSW was measured. We would expect slightly denser plumes to travel further down the slope, but we have no evidence that the water is more dense in 2018 than in 2004.

Cold water less than − 1 °C is also observed at the P2 mooring at Cape Adare during many of the same times that plumes are present at the P3 mooring (Fig. 3, second panel). The two times when cold water appears at the moorings are near the spring and autumn equinoxes when the solar declination is minimum and tidal velocities are weaker (Fig. 3, third panel). However, there are other times when cold water appears periodically, particularly from November through February after weak tidal velocities.

The correspondence between low tidal velocities and appearance of dense water suggests a reduction of tidal mixing in the Drygalski Trough may allow dense water to escape. To investigate the potential tidal control, we created tidal velocities using the timing of the cold periods at the mooring and the observed velocities at mooring G in the Drygalski Trough. These reconstructed tidal velocities (Fig. 3, fourth panel) capture the weaker tides during equinoxes. They also show spring-neap cycles with weaker velocities between November and February, when the diurnal tides are counteracted by the low frequency tides and fewer periods of stronger tidal flow when the two are acting together between May and October.

We examine a relationship between the tidal velocities and the outflow using the inverse of the tidal velocity. Although this scaling approximates a linear drag law^30,31^, ${{\varvec{\tau}}}_{{\varvec{b}}}={\varvec{\rho}}{{\varvec{C}}}_{{\varvec{D}}}\left|{{\varvec{u}}}_{{\varvec{t}}{\varvec{i}}{\varvec{d}}{\varvec{e}}}\right|\boldsymbol{ }{{\varvec{u}}}_{{\varvec{o}}{\varvec{u}}{\varvec{t}}}$, the role of the non-linear terms in the mean momentum balance may be complex^17^ and we do not attempt to account for it. Therefore, the scaling should be regarded as a simple way to account for the regulation of the outflow by the tides and as a starting point for future investigation. Using the inverse of the tidal velocity magnitude (Fig. 3, bottom panel) we find cold water would be released in several pulses around the equinoxes and also in intermittent pulses when tidal velocities are weak after the September equinox.

**Figure 5:**
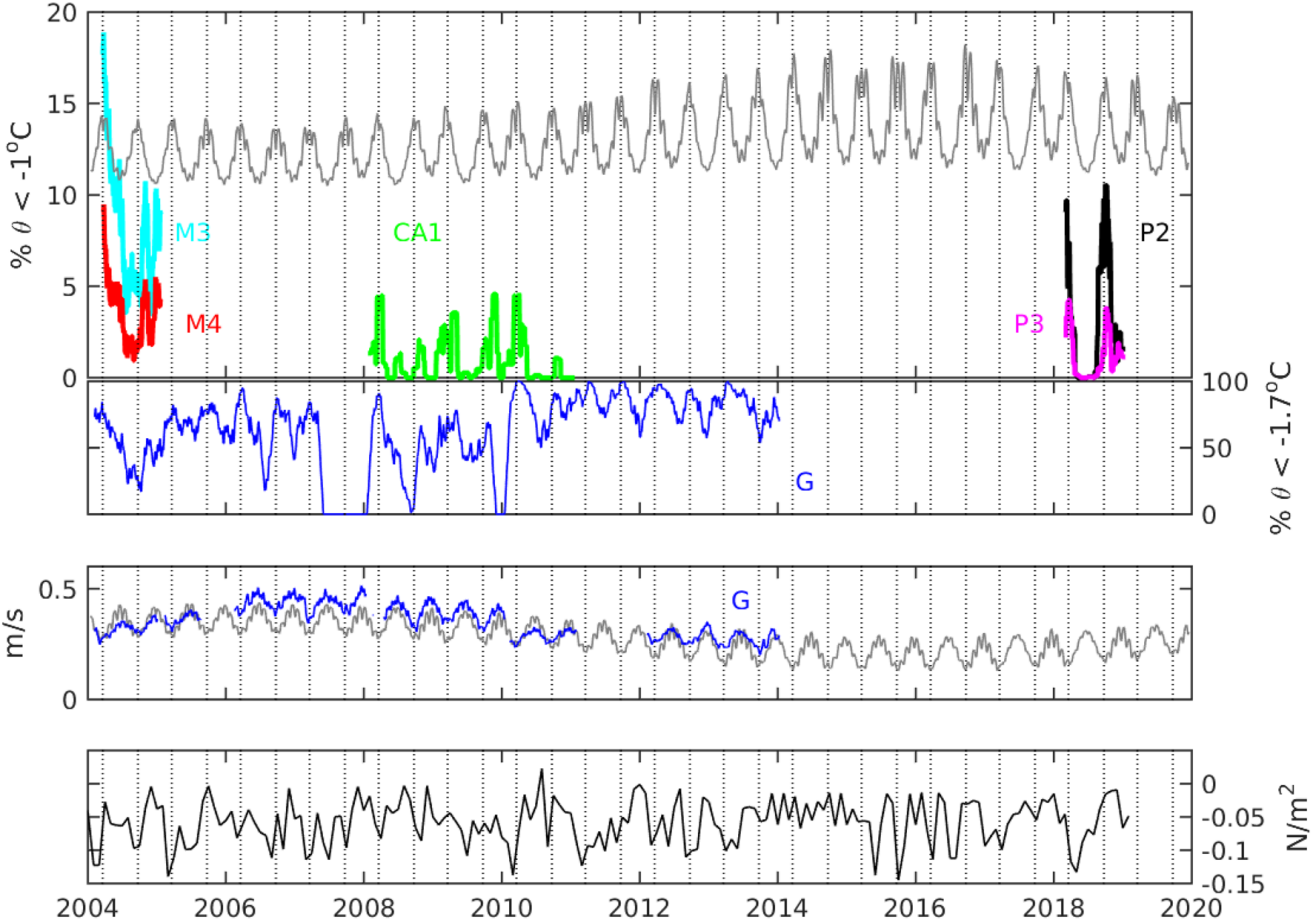
The upper panel shows the percentage of time water less than − 1 °C was measured at the bottom sensor of each mooring every month. The gray line is |u_eq_|^−1^ with arbitrary offset and scaling. The second panel shows the percentage of time water less than − 1.7 °C was measured at the bottom sensor of Mooring G in the Drygalski Trough. The third panel shows the magnitude of the velocity, averaged over a month, at Mooring G with the gray line showing the magnitude of u_eq_ averaged over a month shifted downward by 0.15 m/s. The bottom panel shows the along-slope wind stress in the central Ross Sea, where negative values indicate a greater wind stress towards the west along the shelf. The vertical lines show the times of the equinoxes.

We used the RSO, CALM and AnSlope time series to further investigate the role of the wind and tides over multiple years. Cold water appears at the bottom sensors of all the moorings in March and April with a second period of cold water later in the year (Fig. 5). These periods of cold water measured at all the moorings often line up with the equinoxes, when tidal energy due to the solar diurnal tides is minimum (Fig. 5, top panel), and the alignment is particularly striking during the RSO experiment (P2 and P3). Tidal velocities at the Drygalski Trough Mooring G also show clear minima at the equinoxes and the periods of low tidal energy correspond to the presence of colder temperatures at the mooring (Fig. 5, second panel). The equilibrium tidal velocities (Fig. 5, third panel, gray line) show the 18.6 years lunar declination cycle, increasing to major lunar standstill in 2006 followed by a decrease to minor lunar standstill, when the moon’s range of declinations is minimum in 2015. The velocities at mooring G also show the cycle in lunar declination (Fig. 5, third panel, blue line). Strong velocities correspond with less cold water at the bottom-most sensor in the Drygalski Trough and weaker velocities correspond with more cold water (Fig. 5, third and fourth panels). Some of this variation is likely due to sensor placement, however vigorous tidal mixing is likely to be a factor setting the water properties of the HSSW exiting the trough, with colder water leaving the trough when weaker tidal mixing reduces the amount of warmer CDW being mixed into the dense water near the bottom.

We also take the inverse of the equilibrium tide to create a time series of expected release of dense water from the Drygalski Trough (Fig. 5, grey line in the upper panel). The tidal velocities explain the release of dense water at equinoxes as well as the increase in cold water during 2018 at P2 compared to the 2008–2011 CA1 deployments. The reconstructed tide also reproduces the asymmetry in the occurrence of dense water between the equinoxes: between the March and September equinoxes there is very little release of dense water because the low frequency tide is adding to the diurnal tide, but between the September and March equinoxes there is a “leakage” of dense water due to the low frequency tide periodically counteracting the diurnal tide and reducing the velocity. These features are evident in the moored observations at Cape Adare, where both the P2 and CA1 moorings measure less cold water between March and September compared to the period between the September and March equinoxes (Figs. 4 and 5).

Several of the characteristics of the dense water release cannot be explained by the tides. The AnSlope moorings, M3 and M4, show the release of dense water on the March 2004 equinox, but a release of dense water after the September equinox. Similarly, several peaks in the occurrence of cold water at CA-1 lag the minimum in tidal velocity. It is possible that advection within the Ross Sea sets an additional time scale for the dense water to move towards the shelf break after the tidal mixing weakens. It is also possible that other factors influence the release of dense water through the year, such as the winds. We could find no correspondence between the local wind stress near Cape Adare and the Drygalski Trough or the wind stress curl in the Ross Gyre. However, there is a correspondence between cold water at the moorings and the along-slope wind stress in the central Ross Sea, the region used to investigate the winds in the CALM experiment^11^ (Fig. 5, bottom panel). The correspondence we find is for more cold water when along-slope winds are stronger in the direction of the polar easterlies, which should tend to move the front onshore. This result is opposite to relationship found in the CALM study^11^ because the wind products have changed markedly between our studies. The role of the winds and the potential interplay of winds and tides on the outflow are questions deserving further study.

## Discussion

The two co-located mooring deployments at Cape Adare show dense water appearing twice a year with highest salinities measured in March. The analysis of water properties shows that both pulses of dense water near the bottom at Cape Adare in 2018 can be explained as an increase in HSSW water from the Drygalski Trough. Using the seasonal cycle in the salinity of HSSW from the Drygalski Trough^22^, the dense water at Cape Adare consists of 10–36% HSSW with two peaks, one in February/March and the other in September/October. When dense water is observed at Cape Adare, dense plumes of HSSW from the Drygalski Trough also appear at RSO-P3 on the slope near the trough.

The pulses of dense water at Cape Adare during the RSO experiment are aligned with the equinoxes when solar declination is minimum. A semi-annual variation was also noted in the appearance of dense water at Cape Adare during the CALM experiment^11^. Plumes of dense water were most prevalent between November and May during the AnSlope experiment^9^, with the spring-neap cycle also modulating the volume and properties of dense water^20^. We find the diurnal and low frequency tides can explain the semi-annual occurrence of cold water at Cape Adare as well as the bursts of dense water that appear between the September and March equinoxes. We also suggest the 18.6 years modulation of lunar declination creates more pronounced pulses of dense water at the equinoxes in 2018 compared to the equinoxes in 2008 through 2010. An inverse relationship with the tidal velocity suggests the years around the lunar minimum in 2015 (and every 18.6 years) are the most favorable for dense water outflow from the western Ross Sea. It is possible that the tides are also modulating the dense outflows from the other troughs in the Ross Sea.

The increase in salinity off Cape Adare from 2008 to 2010–2018 is consistent with the observed changes in water properties in the Ross Sea. Salinity decreased in the Drygalski Trough from 2008 until 2014, followed by a more rapid increase from 2014 to 2018^7^. The CALM observations show a slight decrease of salinity of 0.007/year^11^, also consistent with the observations in the Ross Sea. The recent salinity increase has been linked to increase in sea ice production in the Ross Sea^32^.

Water properties in the Ross Sea depend on the exchange of dense water flowing out with an inflow of CDW across the slope. Simulations show ~ 50% of the CDW around the Antarctic crosses the slope into the Ross Sea^33^. CDW is found far south in the Drygalski Trough^21^ and the appearance of CDW at Mooring G is strongly modulated by the semi-annual tides^22^. Simulations also show the inflow of CDW and outflow of dense water are co-located in the troughs of the Ross Sea^13,33^. Therefore, export of dense water and the inflow of CDW are dynamically linked, as regional process studies also suggest^17^. Here, we suggest tidal flows may be modulating the exchange over a range of frequencies, as occurs in shallow systems^34^, in addition to internal hydraulic controls found in deeper constrictions^35^. However, more work is needed to reconcile the scaling of bottom stress that we have used with the more complex dependence of the outflow on the tides discussed in process simulations^15,17^. Nevertheless, the regularity of the tides suggests we may be able to predict future exchange of CDW and HSSW across the Ross Sea shelf break and estimate exchange in the past.

## Methods

### Ross sea outflow (RSO) experiment

A mooring (RSO-P2) was placed at Cape Adare (Fig. 1) from 19 February, 2018, to 17 January, 2019, in 1740 m of water to measure water properties and bottom water velocities at the same location as the CA-1 mooring from the CALM study^11^. The mooring extended over the lower 478 m of the water column and was instrumented with sensors at similar depths to the CALM mooring (Table 1) to capture the benthic layer flow, extending the CALM time series. Another mooring (RSO-P3), with the same configuration of instruments, was placed on a similar isobath on the slope north of the Drygalski Trough (Fig. 1 and Table 1) to measure water properties flowing towards Cape Adare from east of the Drygalski Trough.

**Table 1:** Instrumentation, depth, location and deployment dates for the P2 and P3 moorings.

Mooring	Distance above bottom (m)	Salinity	STD	Potential temperature (°C)	STD	Potential density	STD	Speed (m/s)	STD
**P2**									
− 71.4601	478	34.692	0.006	0.215	0.146	36.238	0.015		
172.3024	477							0.13	0.07
1740 m	300	34.694	0.008	− 0.074	0.155	36.270	0.018		
2018/2/19	177			No data					
2019/1/17	48	34.693	0.015	− 0.513	0.226	36.314	0.027		
	20							0.47*	0.14*
**P3**									
− 71.9181	453	34.697	0.005	0.412	0.141	36.219	0.013		
172.9265	452							0.19	0.01
1715 m	301	34.651	0.007	0.177	0.189	36.236	0.017		
2018/2/20	217			No data					
2019/1/17	48	34.645	0.010	− 0.275	0.268	36.277	0.027		
	22							No data	

The mean and standard deviation of properties from each sensor are given. Three instruments did not return data and the lowest current meter on the P2 mooring collected data for only 1 month (denoted by an asterisk).

Most sensors returned a complete time series. However, the batteries in the bottom current meters on both moorings did not function properly: as a result, there are no near-bottom velocities at P3 and only during the first deployment month at P2.

During the February 2018 voyage, three hydrographic sections were carried out perpendicular to the slope (Fig. 1) to measure the temperature and salinity along the slope. The section off Cape Adare intersects the location of RSO-P2 and was completed first, followed by the section oriented nearly north to south at the mouth of Drygalski Trough that contains the RSO-P3 mooring location. A third section was taken across the slope near Cape McCormick. The deepest casts in this hydrographic section sampled a dense plume of HSSW from the Drygalski Trough (Fig. 1 and see Supplementary Fig. S1). The plume was not present in the section further south near the Drygalski Trough: it may be that the dense plume was not exiting the trough when the section further south was completed a day earlier or it may be because the plume was exiting at shallower depths than were measured in the section.

### CALM, AnSlope and MORSea experiments

Moored observations from several previous experiments were used in this study. The mooring CA-1 from the CALM experiment was deployed between January 2008 and January 2011^36^. Measurements from this mooring were compared to those at RSO-P2 and consist of temperature at 43 m, 298 m and 460 m above bottom, salinity at 43 m and 460 m, and velocity at 476 m above bottom. Moored observations from the AnSlope experiment during 2004^9^ were also used to examine the differences in dense plumes between the RSO-P3 mooring and to investigate the timing of the dense plumes relative to the tides. The temperature and salinity from the sensors 10 m above bottom on the M3 (691 m), M4 (984 m), and M5 (1749 m) moorings were examined. Hydrographic data collected at Cape Adare in 2004 during the AnSlope experiment was also compared to the hydrographic section collected in 2018.

The bottom water properties and flow within the Drygalski Trough were compared to the measurements on the slope using the observations at the Marine Observatory of the Ross Sea (MORSea) mooring G maintained over 10 years near 72.4° S, 173° E in ~ 520 m water depth^22^. Observations of temperature and velocity from 2004 to 2014 from the bottom-most sensors, which range in depth from 8 to 70 m above bottom, were used along with salinity measured from 2005 through 2008^22^ (see Supplementary Fig. S4).

### Analysis of seasonal and interannual variability

To examine the seasonal and interannual variations in water properties and currents, the moored observations from the RSO-P2 and CALM CA-1 moorings were filtered over 31 days using a cosine filter. Blow down of the RSO-P2 mooring was minimal (98% of the time the upper sensor was within 20 m of the minimum pressure and the lower sensors were within 6 m of the minimum pressure). Therefore, we averaged the sensor data over the entire time period to obtain mean values. Averages over the spring-neap cycle were performed with a 31-day cosine filter.

### Identification of dense water at the moorings

Dense water is observed as short pulses at both moorings, identified by a rapid decrease in temperature and increase in salinity, followed by a slower recovery to background values. Cold plumes were identified in the moored records, following the AnSlope analysis^9^, by finding any time with potential temperature less than − 1 °C. The presence of HSSW was identified when salinity was greater than 34.75. The hours per week and per month these properties were present were also calculated at each sensor.

Plume events were also identified as events when the difference between the maximum and minimum temperature over any hour was greater than 0.5 °C and the minimum temperature was also less than − 1 °C. At the RSO-P3 mooring there were 74 such instances (see Supplementary Fig. S3). At the RSO-P2 mooring, changes in temperature were less sudden and tended to coincide with changes in the tidal velocity and, as a result, only one event fit the definition.

### Tidal analysis

To test whether cold water measured at the moorings at Cape Adare is released when tidal velocities in the Drygalski Trough are weak, we examined the relationship between the occurrence of cold water and the tides. The equilibrium tidal potential ($\zeta$) due to any celestial body has a low frequency component (${\zeta }_{0}$), a diurnal component (${\zeta }_{1}),$ and a semidiurnal component (${\zeta }_{2})$ which can be related to known astronomical variables^37^:

$$\zeta = \zeta_{0} + \zeta_{1} + \zeta_{2}$$

$$\zeta = \frac{{mR^{4} }}{{4Mr^{3} }}\left[ {\left( {3\;sin^{2} \;\theta - 1} \right)\left( {3\;sin^{2} \;\delta - 1} \right) + 3\;sin\;2\theta \;sin\;2\delta \;cos\;\Delta \lambda + 3\;cos^{2} \;\theta \;cos^{2} \;\delta \;cos\;2\Delta \lambda } \right]$$

where the mass and radius of the Earth are M and R, the mass of the celestial body is m, the distance between the Earth and the body is r, the latitude on the Earth is $\theta$, the declination of the body is $\delta$ and $\Delta \lambda$ is the changing longitude due to the revolution of the Earth.

We calculated the solar and lunar equilibrium tides using ephemerides from Jet Propulsion Laboratory Horizons Web-Interface^38^. At high latitudes, the semidiurnal component is small and varies little with declination; the diurnal and low frequency equilibrium tides are much larger. In a channel, the tidal velocity is proportional to the tidal potential but multiplied by factors due to propagation and resonance^39^. We therefore constructed the tidal velocity from the low frequency and diurnal components and found the coefficients that best fit the observations:

$$u_{eq} = A\frac{{\zeta_{0} }}{{10^{9} }} + B\frac{{\zeta_{1} }}{{10^{9} }} + C$$

where now the components ${\zeta }_{0}$ and ${\zeta }_{1}$ are the sum of the solar and lunar tidal potentials. We found the relationship between A, B and C by finding the best fit to times when the velocity is near zero in the Drygalski Trough mooring. We fit to times when ${u}_{eq}=0$ to simplify the fit and constrain it to times when mixing is weak. Taking all times when the weekly average of the velocity magnitude is less than 0.2 m/s gives [A, B, C] = [0.71, − 1, 0.76]. Adjusting the velocities for the different heights of the sensors using a log layer profile gives [A, B, C] = [0.87, − 1, 0.95]. We made an additional independent estimate by finding the best fit to times when cold water was most prevalent at each mooring: we assume ${u}_{eq}=0$ for weeks when the number of cold hours is within 20% of the maximum value at each mooring and found [A, B, C] = [1.16, − 1, 1.18] (see Supplementary Fig. S5).

Since the aim is to capture the main characteristics of the tides, we chose round numbers, [A, B, C] = [1, − 1, 1], and constructed the equilibrium tide, ${u}_{eq}$, over the whole time period. We also compared the presence of cold water at the moorings with the inverse of the tidal velocity, ${{|u}_{eq}|}^{-1}$, which we used as a scale for the reduction of bottom drag and potential release of HSSW from the Drygalski Trough.

### Wind analysis

We investigated whether variability of the regional winds may be related to flow and water properties from the moored observations using monthly mean wind stress from the Fifth generation of the European Centre for Medium-Range Weather Forecasts atmospheric reanalysis (ERA-5, 1/4° × 1/4° resolution). We averaged wind stress over the same region used in the CALM experiment, 70°–75° S and 175° E–175° W, and rotated the coordinate system 40° to follow the direction of the slope in the central Ross Sea at 175^o^W as done previously^11^. We also examined the wind stress curl and averaged it over the Ross Gyre region from 170°E to 130°W and 65°–75° S, and examined local wind stress along the slope at both the Drygalski Trough and Cape Adare.

### Transport from hydrography

The along-slope transports were calculated from the hydrographic sections in February 2018. Dynamic heights were calculated from temperature and salinity profiles (Fig. 1 and see Supplementary Figs. S1 and S2) and geostrophic shear estimated between each pair of profiles across each section. A mean dynamic topography (CLS-CNES2013)^40^ was interpolated to the location of each profile and the surface geostrophic velocity calculated between each profile and added to the shear from the hydrography to obtain the total geostrophic velocity. The velocities were then integrated within density ranges to find the transport of dense water. We used neutral densities greater than 28.27 to define the dense water associated with Ross Sea bottom water. We also found the transport of AABW by integrating velocities of water with properties between − 0.75 < θ < 0.24 and 34.68 < S < 34.72^41,42^ in depths over 1000 m. The along-slope transport was calculated in the same way from an identical section at Cape Adare collected in March, 2004.

## Supplementary Information

Supplementary Information.

## Data Availability

The hydrographic data are available on the World Ocean Database and the moored data from the MORSea Project website*.*
