# Supplementary material for: The role of tides in bottom water export from the western Ross Sea
Source: Sci Rep. 2021 Jan 26;11:2246. doi: 10.1038/s41598-021-81793-5 (PMC7838284; doi:10.1038/s41598-021-81793-5)
Supplement: Supplementary file 1 — Supplementary Information. [file 41598_2021_81793_MOESM1_ESM.docx]

**The role of tides in bottom water export from the western Ross Sea**

**Melissa M. Bowen, Denise Fernandez, Aitana Forcen-Vazquez, Arnold L. Gordon, Bruce Huber, Pasquale Castagno, and Pierpaolo Falco**

**Supplementary Material**


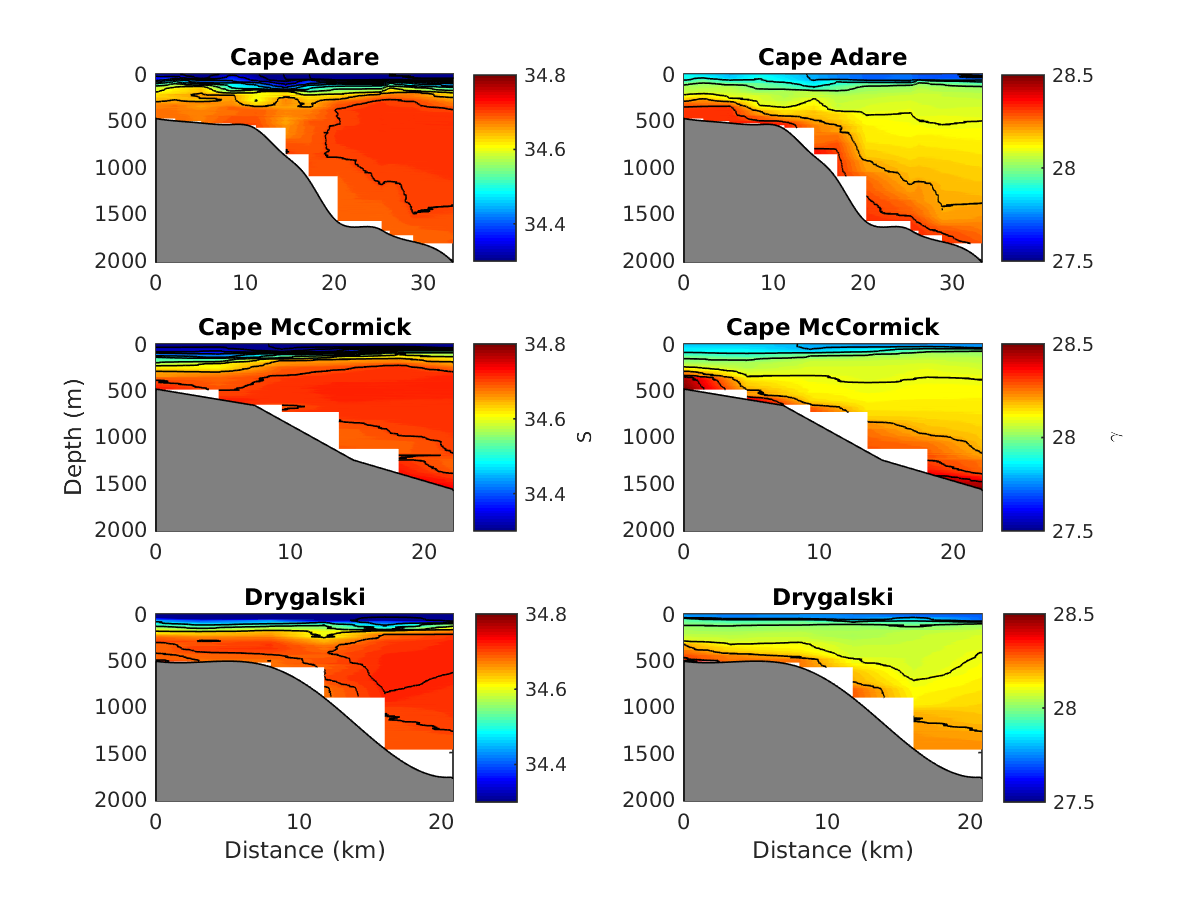


Figure S1: Salinity (left) and neutral density (right) from the three sections across the slope at Cape Adare (top), Cape McCormick (center) and near the Drygalski Trough (bottom). (Figure produced in Matlab R2015a https://au.mathworks.com/products/matlab.html.)


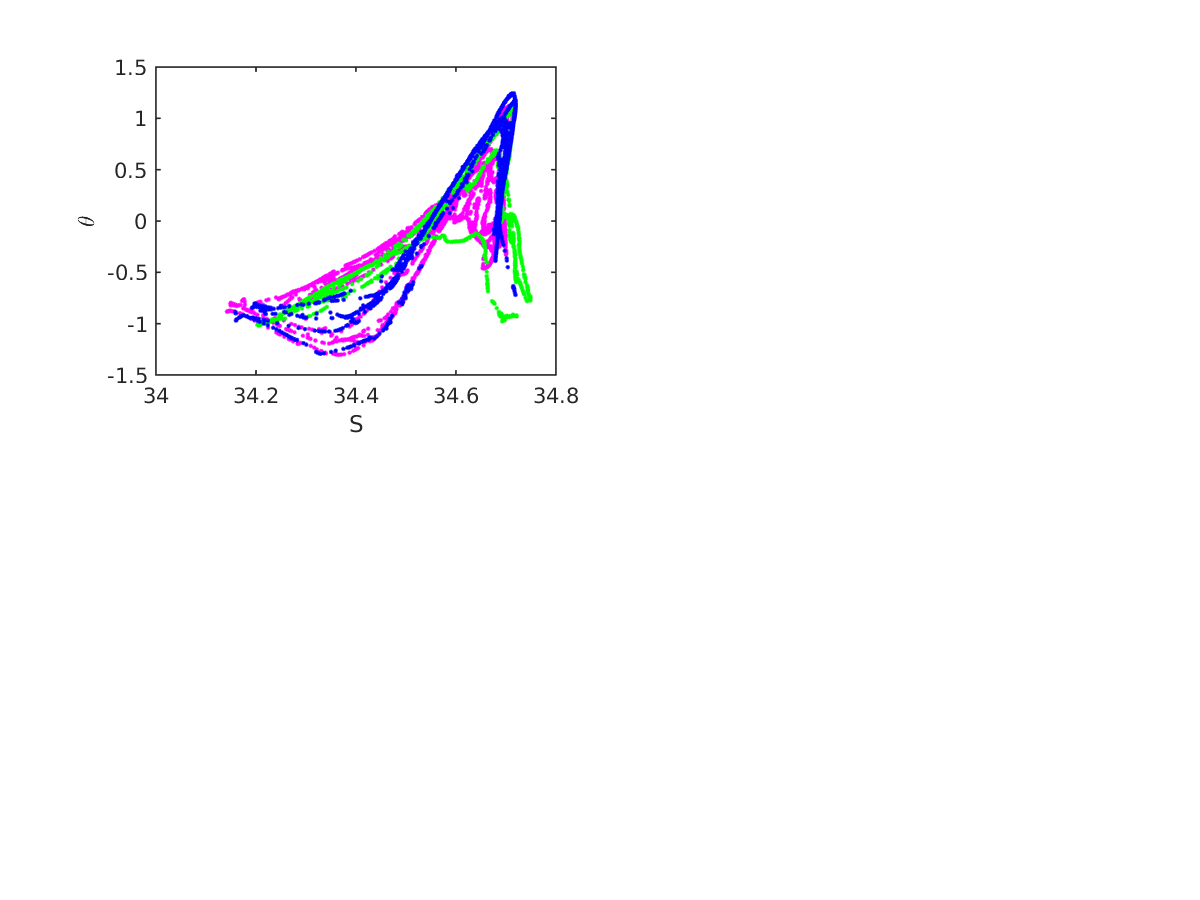


Figure S2: θ-S plot from the three hydrographic sections. Data from the Cape Adare section is in pink, Cape McCormick in green and the section on the slope near the Drygalski Trough in blue.


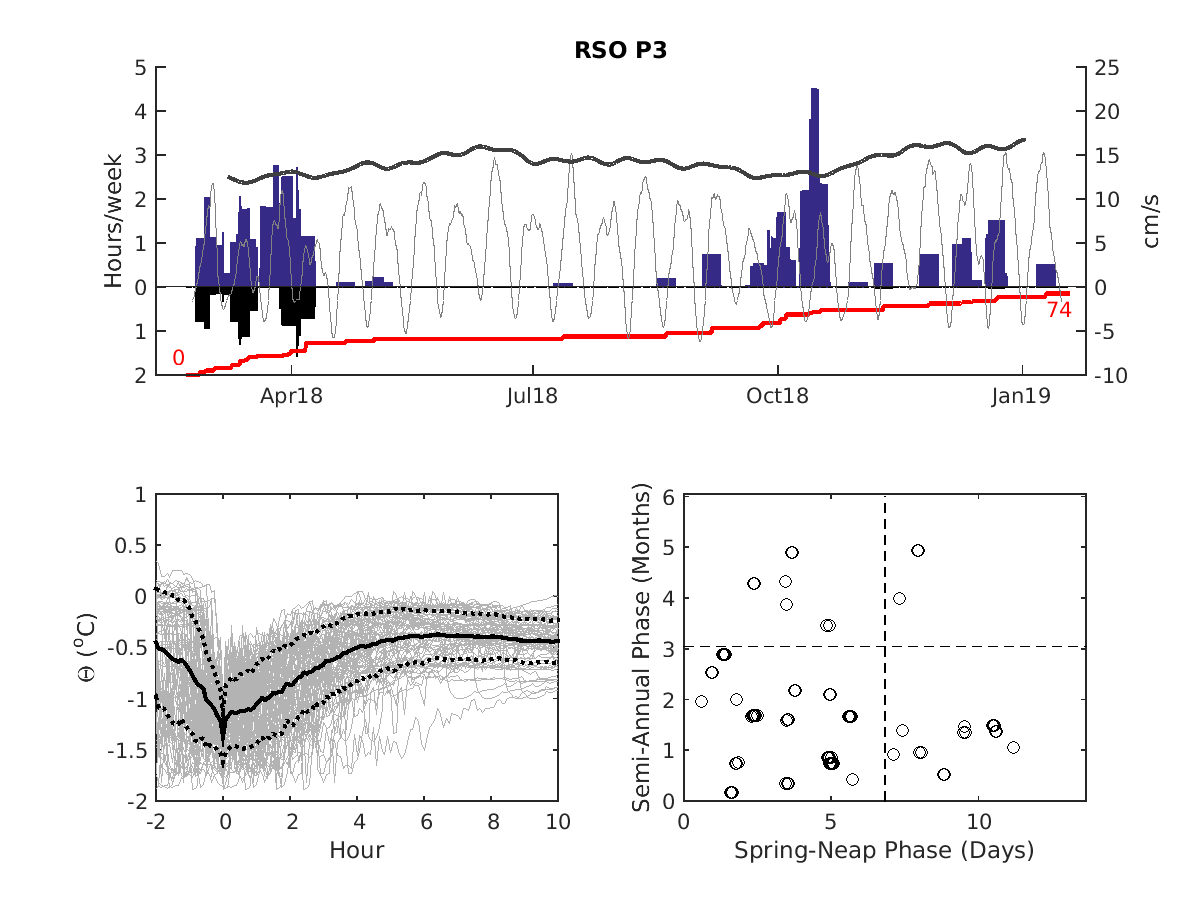


Figure S3: Potential temperature of the 73 plume events identified at the bottom sensor of the P3 mooring. The temperature has been lined up so the change in water properties coincides with the origin on the time axis. The solid line shows a composite of the events and the dashed line the standard deviation.


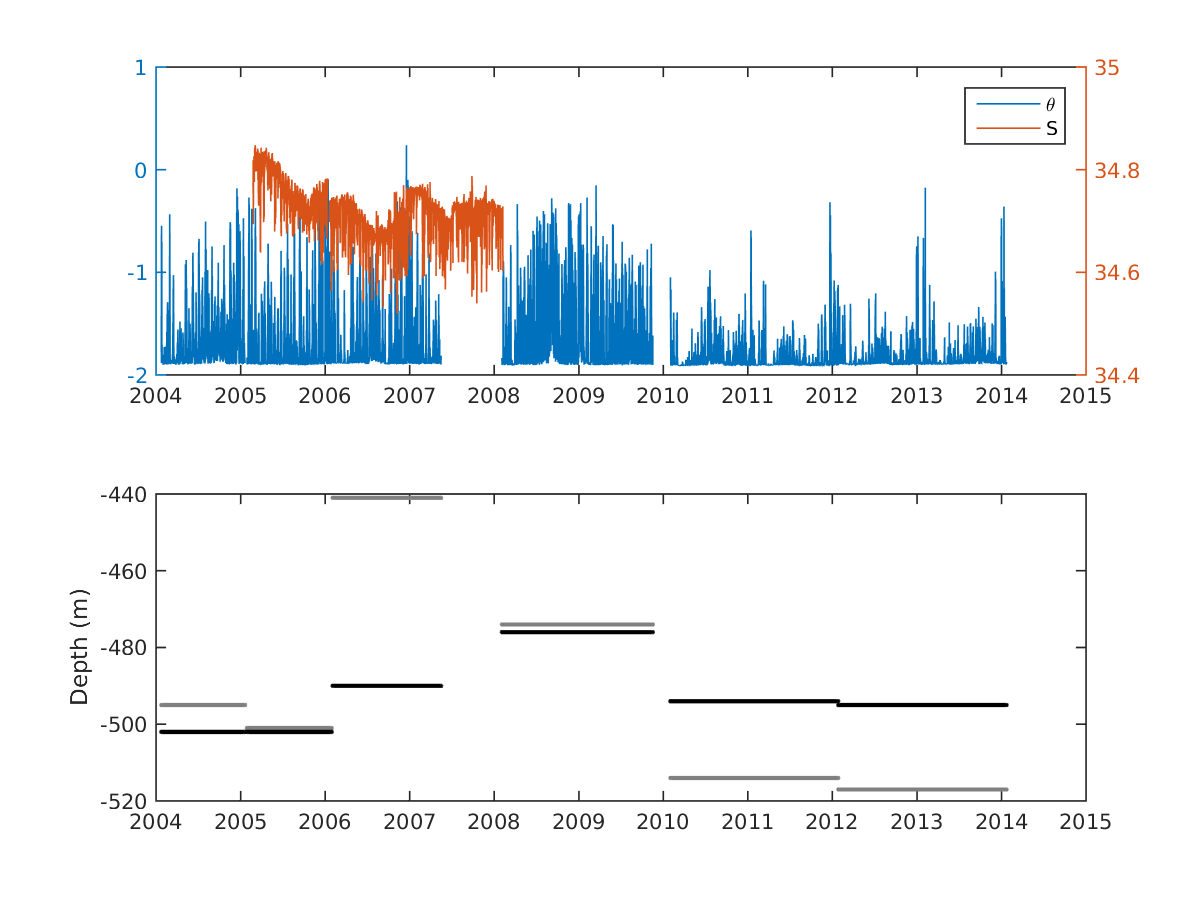


Figure S4: The temperature and salinity from the near bottom sensor of Mooring G (upper panel) and the depth of the temperature and salinity measurement (black lines) and the depth of the near bottom velocity measurement (gray line).

Table S1: Potential temperature and salinity of HSSW from the lowest sensor at Mooring G, CDW from the RSO hydrography and AABW from the P3 mooring.

| Month | HSSW(θ) | HSSW (S) | CDW (θ) | CDW (S) | AABW (θ) | AABW (S) |
| --- | --- | --- | --- | --- | --- | --- |
| Jan | -1.75 | 34.73 | 1.27 | 34.72 | -0.32 | 34.68 |
| Feb | -1.81 | 34.75 | 1.27 | 34.72 | -0.44 | 34.68 |
| Mar | -1.86 | 34.75 | 1.27 | 34.72 | -0.39 | 34.68 |
| Apr | -1.85 | 34.75 | 1.27 | 34.72 | -0.39 | 34.68 |
| May | -1.84 | 34.74 | 1.27 | 34.72 | -0.23 | 34.68 |
| Jun | -1.81 | 34.73 | 1.27 | 34.72 | -0.23 | 34.68 |
| Jul | -1.77 | 34.71 | 1.27 | 34.72 | -0.09 | 34.68 |
| Aug | -1.77 | 34.70 | 1.27 | 34.72 | -0.14 | 34.68 |
| Sep | -1.77 | 34.70 | 1.27 | 34.72 | -0.37 | 34.67 |
| Oct | -1.83 | 34.71 | 1.27 | 34.72 | -0.44 | 34.67 |
| Nov | -1.80 | 34.71 | 1.27 | 34.72 | -0.27 | 34.67 |
| Dec | -1.73 | 34.71 | 1.27 | 34.72 | -0.22 | 34.67 |


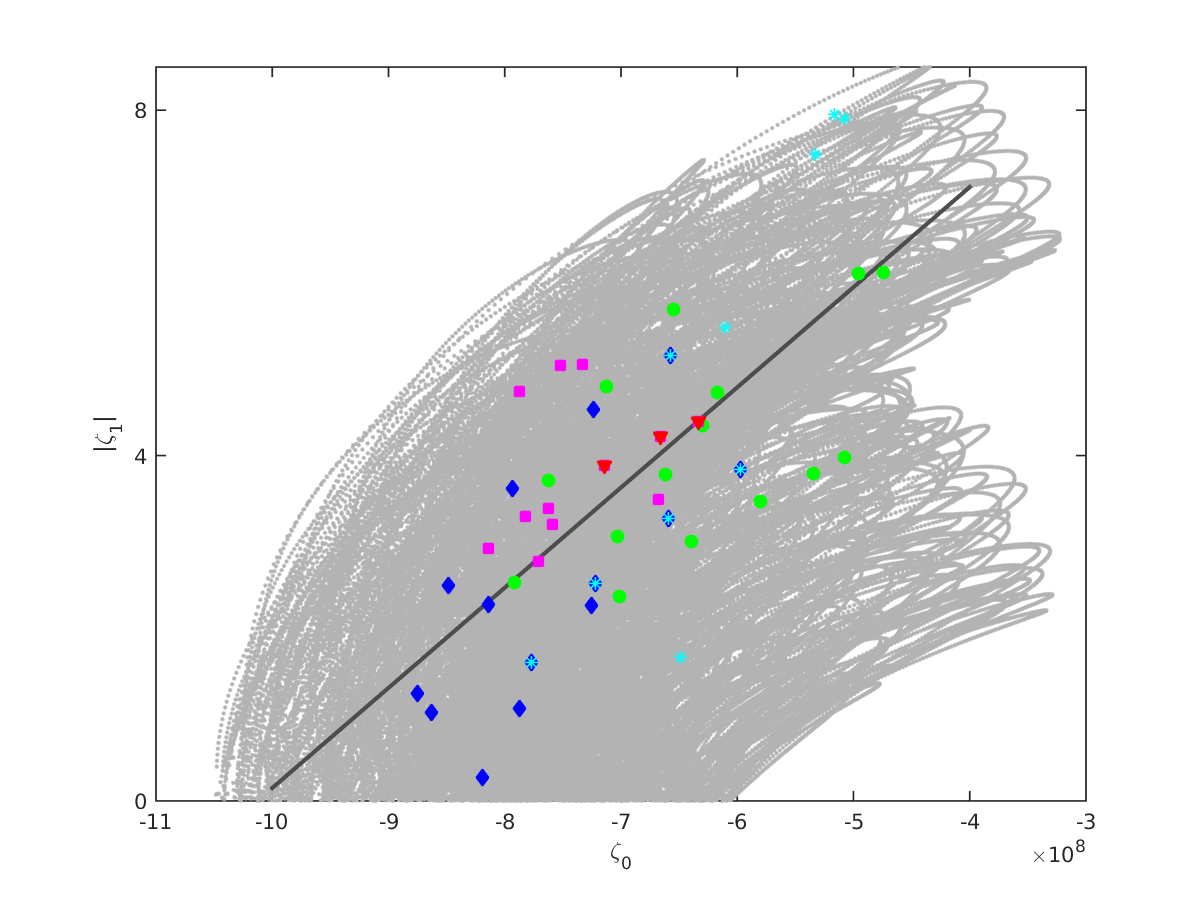


Figure S5: The colored dots show the diurnal and low frequency tides for the weeks when the most cold water was present at each mooring (weeks when occurrence of cold water was within 20% of the maximum value at the mooring) for P2 (pink), P3 (red), CA1 (green), M3 (dark blue) and M4 (light blue). The grey dots show the range of values over the whole time period from 2004 through 2018. The line shows the best fit from the mooring values.

*Table S2. Percentage of CDW, AABW at the P3 mooring and HSSW at MORSea G that combine to give the water properties at the P2 mooring at Cape Adare each month. The bottom sensors on all the moorings were used to estimate the water properties.*

| Month | %CDW (CTDs) | %AABW (P3) | % HSSW (MORSea G) |
| --- | --- | --- | --- |
| Jan | 28 | 46 | 26 |
| Feb | 27 | 37 | 36 |
| Mar | 26 | 41 | 34 |
| Apr | 24 | 49 | 28 |
| May | 20 | 62 | 18 |
| Jun | 11 | 79 | 10 |
| Jul | 0 | 90 | 10 |
| Aug | 2 | 88 | 11 |
| Sep | 10 | 69 | 22 |
| Oct | 14 | 63 | 23 |
| Nov | 12 | 68 | 19 |
| Dec | 20 | 56 | 24 |
